# “An on-going individual adjustment”: a qualitative study of midwives’ experiences counselling pregnant women on physical activity in Sweden

**DOI:** 10.1186/1471-2393-14-343

**Published:** 2014-09-30

**Authors:** Maria Lindqvist, Ingrid Mogren, Eva Eurenius, Kristina Edvardsson, Margareta Persson

**Affiliations:** Department of Clinical Sciences, Obstetrics and Gynaecology, Umeå University, Umeå, Sweden; Departments of Public Health and Clinical Medicine, Epidemiology and Global Health, Umeå University, Umeå, Sweden; Judith Lumley Centre, La Trobe University, Melbourne, Australia; School of Health and Social studies, Dalarna University, Falun, Sweden; Department of Nursing, Umeå University, Umeå, Sweden

## Abstract

**Background:**

In Sweden, midwives play prominent supportive role in antenatal care by counselling and promoting healthy lifestyles. This study aimed to explore how Swedish midwives experience the counselling of pregnant women on physical activity, specifically focusing on facilitators and barriers during pregnancy. Also, addressing whether the midwives perceive that their own lifestyle and body shape may influence the content of the counselling they provide.

**Methods:**

Eight focus group discussions (FGD) were conducted with 41 midwives working in antenatal care clinics in different parts of Sweden between September 2013 and January 2014. Purposive sampling was applied to ensure a variation in age, work experience, and geographical location. The FGD were digitally recorded, transcribed verbatim, and analyzed using manifest and latent content analysis.

**Results:**

The main theme– *“An on-going individual adjustment”* was built on three categories: *“Counselling as a challenge”; “Counselling as walking the thin ice” and “Counselling as an opportunity”* reflecting the midwives on-going need to adjust their counselling depending on each woman’s specific situation. Furthermore, counselling pregnant women on physical activity was experienced as complex and ambiguous, presenting challenges as well as opportunities. When midwives challenged barriers to physical activity, they risked being rejected by the pregnant women. Despite risking rejection, the midwives tried to promote increased physical activity based on their assessment of individual needs of the pregnant woman. Some participants felt that their own lifestyle and body shape might negatively influence the counselling; however, the majority of participants did not agree with this perspective.

**Conclusions:**

Counselling on physical activity during pregnancy may be a challenging task for midwives, characterized by on-going adjustments based on a pregnant woman’s individual needs. Midwives strive to find individual solutions to encourage physical activity. However, to improve their counselling, midwives may benefit from further training, also organizational and financial barriers need to be addressed. Such efforts might result in improved opportunities to further support pregnant women’s motivation for performance of physical activity.

## Background

In Sweden, almost all pregnant women regularly visit midwives in antenatal care (ANC). One of the aims of ANC services in Sweden is to achieve “good sexual and reproductive health for the whole population”, which is in agreement with the WHO definition of sexual and reproductive health [1]. The ANC services are free of charge and organized in 43 Maternal Health Care Areas (MHCA), mainly corresponding to Swedish counties (personal communication). Each MHCA is led by a consultant obstetrician and a consultant midwife who have the responsibility to develop medical guidelines, in-service training, and evaluation of provided health services at the ANC. More than a third of the Swedish ANC centers are currently organized within primary health care, a quarter are organized as free-standing units, another quarter are private clinics, and the rest are organized as part of departments of obstetrics and gynaecology. Midwives working in ANC provide medical support for all fertile women as well as medical surveillance during pregnancy. The professional associations of obstetricians and midwives have issued national guidelines for surveillance during pregnancy [1]. These guidelines specify eight to ten ANC visits for uncomplicated pregnancies. The ANC surveillance programme aims to identify risk factors during pregnancy that could result in complications for the mother or foetus. The midwife also provides counselling on healthy lifestyle [1].

The content and quality of the relationship established between the midwife and the pregnant woman is central for successful health care. Effective communication not only increases the quality of maternity care, but also ensures clinical safety [2–5]. In addition, trust and confidence are significant components with respect to the relationship between caregivers and patients [6]. The supportive role of the midwife is prominent as is her mission to promote a healthy lifestyle during pregnancy. Furthermore, an individual assessment of the woman’s psychosocial situation is part of the midwife’s supportive and counselling role [1]. Swedish midwives apply different strategies, such as being a caring companion or acting as a medical guardian, when advising pregnant woman and her partner to promote healthy behavior and to protect the relationship. These strategies depend on whether the pregnancy is normal or complicated [5, 7].

The Swedish national guidelines recommend 30 minutes of physical activity per day during normal pregnancy [1]. The current national recommendations, based on the international WHO recommendations, suggest that adults 18 to 64 years of age, pregnant women included, should perform at least 150 minutes per week of moderate intensity aerobic physical activity or at least 75 minutes per week of vigorous intensity aerobic physical activity or a combination of these. In the majority of cases, physical activity is a safe and beneficial component of a healthy pregnancy and does not cause an increased risk of adverse pregnancy outcome [8]. Regular physical exercise can maintain or improve fitness during pregnancy and may reduce adverse pregnancy outcomes, such as gestational diabetes mellitus and preeclampsia [8, 9].

As reported by pregnant women, barriers for performing physical activity during pregnancy include lack of time and motivation, increased body size, pain, and other physical barriers [10, 11]. The wellbeing of the foetus can motivate pregnant women to perform physical activity and strive for a healthy lifestyle [11, 12]; however, pregnant women may receive conflicting information about physical activity from health care providers and other health professionals [11]. Furthermore, previous studies have also shown that pregnant women experience lack of information regarding physical activity, advice on appropriate gestational weight gain, and physical exercise during pregnancy. In addition, they often perceive the caregivers’ knowledge as limited [13]. A qualitative study concludes that pregnant women need unambiguous advice regarding healthy lifestyle, diet, and exercise during pregnancy [14]. As a result of these findings, health care providers are demanding specific evidence-based guidelines on physical activity for pregnant woman [11]. Physical activity is associated with very good or excellent self-rated health [15] and well-being, and women who are physically active before pregnancy seem to continue their physically active lifestyle into pregnancy [16].

Few publications have addressed counselling on physical activity during pregnancy. To our knowledge, no study has been conducted that explores how Swedish midwives experience counselling pregnant women on physical activity. This study attempts to fill this gap in knowledge.

### Aims

This study aimed to explore how Swedish midwives experience the counselling of pregnant women on physical activity, specifically focusing on facilitators and barriers during pregnancy. Also, addressing whether the midwives perceive that their own lifestyle and body shape may influence the content of the counselling they provide.

## Methods

A qualitative approach was applied in order to explore the research questions under study. By using focus group discussions (FGD), experiences and opinions of the participants were collected. Swedish ANC centers were selected to represent differences related to age, size of city, MHCA, demography, socio-economy, coastland, and inland. To be included in the study, the participant had to work as a midwife in an ANC exclusively. Purposive sampling was applied when inviting eligible participants to participate in the study. The study aimed for variety in age, work experience as a midwife, and number of years of employment in an ANC. We aimed for six to eight participants in each FGD in the selected areas. This goal was impossible to fulfill in the most rural areas as midwives worked in small units with few employees or units were located far from each other.

The first author (ML) contacted the consultant midwife in each selected MHCA to distribute the information on the study to the midwives working in the specific MHCA. Thereafter, the consultant midwife provided a list with names of midwives who had been approached, who fulfilled the inclusion criteria, and who were willing to participate to the first author (ML). Before the FGD, ML orally and in writing informed each potential participant about the objectives of the study. Each participant signed a consent form before the FGD started. No eligible participant declined participation in the study. The participants were informed that they could drop out at any time during the study period. The characteristics of the participants are presented in Table 1. Shortly before one of the FGD, three of five participants dropped out due to unexpected work tasks, resulting in only two participants. A majority of the participants (68.3%) reported additional education, such as courses in diet, motivational interviewing, psychology, breastfeeding, pedagogics, and sexology.

**Table 1:** **Characteristics of participants (n = 41) attending the focus group discussions (FGD)**

FGD	(n)	Mean age years	Work experience as midwife	Work experience in ANC
			mean years (range)	mean years (range)
**Northern Sweden**				
FGD 1	2	49.5	17.5 (10-25)	9.5 (4-15)
FGD 2	7	53.2	23.7 (5-35)	12.7 (5-27)
FGD 3	6	52.5	26.7 (2-35)	16.7 (8-27)
**Mid Sweden**				
FGD 4	5	36.5	7.0 (2-9)	2.3 (1-5)
FGD 5	5	58.3	23.0 (5-26)	16.5 (4-24)
FGD 6	9	42.1	16.1 (8-19)	11.9 (3-17)
**Southern Sweden**				
FGD 7	5	48.8	13.8 (2-22)	12.8 (2-18)
FGD 8	2	47.5	17.5 (15-20)	7.5 (2-13)

An interview guide was developed based on ideas from the current literature as well as on the authors’ work experiences. As two of the authors (MP and IM) had significant experience with clinical work in ANC (MP as a midwife and IM as a consultant obstetrician), they understood the organization and the content of the daily work in the ANC. This experience and knowledge was essential to the development of the interview guide. A pilot FGD was conducted including seven midwives currently working in ANC to test the interview guide; this pilot FGD resulted in a few minor revisions of the interview guide. The pilot study was later included in the data analysis with the permission of the participants. The specific topics of the interview guide were the midwives’ experiences of counselling pregnant women on physical activity during pregnancy in relation to:

- Counselling on physical activity in general
- Barriers and facilitators for physical activity
- Counselling native Swedish women
- Counselling immigrant women
- Significance of midwife’s own body shape
- Significance of midwife’s own lifestyle

### Data analysis

The transcribed interviews were analyzed using qualitative manifest and latent content analysis, a systematic approach that analyses what the text says and how the underlying meaning of the text could be interpreted [17]. First, the transcripts were read several times by the first and the last authors (ML and MP) to achieve a sense of the whole and to identify possible content areas. Second, meaning units were identified, condensed, and coded. Third, the codes were compared to identify similarities and differences in content and meaning. During this process, sub-categories and categories were identified. Fourth, the transcripts were then re-read several times to ensure that the categories covered all the aspects of the interviews. During the creation of the categories, the discussion of the findings was mainly performed by ML and MP with input from IM. Later in the analysis, all authors had an opportunity to challenge and discuss the findings until consensus was reached. To validate the findings, the first author arranged a meeting with participants in FGD 4; all but one participant participated. The preliminary results were presented and discussed with the participants. In general, the participants confirmed our findings. During the final steps of the analysis, a theme (i.e., a thread of underlying meanings of the experience under study) emerged [17]. The theme captured how the overall experience of counselling on physical activity was described by the participating midwives.

### Ethical approval

Ethical approval for the study was granted by the Ethical Review Board, Umeå University (Dno. 2012-407-31 M).

### Results

An overview of the theme, categories, and subcategories is presented in Table 2. All quotations used to illustrate the categories are presented in italics and with the focus group number listed after each quotation. The theme revealed – *An on-going individual adjustment –* reflects the midwives on-going need to adjust their counselling approach depending on each woman’s specific situation. Furthermore, counselling pregnant women on physical activity was experienced as complex and ambiguous, presenting challenges as well as opportunities. When midwives challenged barriers to physical activity, they risked being rejected by the pregnant women. Despite risking rejection, the midwives tried to promote increased physical activity based on their assessment of individual needs of the pregnant woman. Some participants reported that their own lifestyle and body shape might negatively influence the counselling; however, the majority of participants did not agree with this perspective.

**Table 2:** **Theme, categories, and sub-categories**

Theme	Category	Sub-category
**An on-going individual adjustment**	**Counselling as a challenge**	• Fighting lack of resources
		• Responding to high-achieving women
		• Responding to perceived barriers to physical activity in pregnant women
		• Responding to the cultural tug-of-war
	**Counselling as walking the thin ice**	• Fearing no success
		• Guarding the relationship
		• Acting upon individual needs
	**Counselling as an opportunity**	• Navigating within cultural traditions
		• Identifying women’s individual facilitators

The presentation of the findings is structured as follows: an initial summary of each category is followed by a presentation of the included sub-categories. Findings are illustrated using quotations.

### Counselling as a challenge

This category comprises four sub-categories: *Fighting lack of resources*; *Responding to high-achieving women; Responding to perceived barriers in pregnant women*; and *Responding to the cultural tug-of-war.* Each sub-category represents aspects of counselling with respect to physical activity experienced as difficult or demanding. These different aspects involve organizational and economical issues that encourage highly active women to rest as well as helping sedentary women to be more active. In addition, these aspects reflect cultural beliefs.

#### Fighting lack of resources

The participants believed that economical cutbacks and organizational changes within the ANCs made their work more difficult, resulting in worse outcomes for the midwives. These changes implied additional work tasks and increased demand for high productivity. As a result, the participants negatively viewed their working conditions; for example, they had less time to fulfill their commitments and less time to provide satisfactory counselling. Experienced participants found that these slowly evolving changes added to their work stress. This demoralizing situation left most participants feeling that they were not providing the best care possible. Furthermore, the economical cutbacks led to decreased possibilities to attend courses or meetings to acquire up-dated information on recommendations and guidelines as well as making it more difficult for them to find inspiration and support for how to best motivate pregnant women to perform physical activity.

*I feel that we’re expected to squeeze in more and more in fewer visits. When I started working, we had maybe 16 visits, but now…We’re expected to fit such a lot into a short time. I would really like to [provide] quality; fewer visits are fine, but you need to have quality…* (Participant, FGD no. 3).

*…what’s more, I don’t know if the money allocated for education has changed or not, but nowadays we have significantly less time for education.* (Participant, FGD no. 8).

#### Responding to high-achieving women

A specifically demanding situation discussed by participants was how to counsel high-achieving pregnant women. These women were in general highly educated, of high socio-economical status, used to accomplishing goals, and to a high extent physically active. The participants perceived this group of pregnant women as generally self-confident, fit, and professionally skilled, but at the same time having problems evaluating their individual needs during pregnancy. As the midwives described that many of these women were physically active at a high intensity level, a more appropriate level of physical performance (i.e., a decreased level of physical activity) was perceived as essential to improve the wellbeing of these women during pregnancy. However, counselling these women to reach a more suitable level of physical activity was perceived as very challenging:

*They have good self-confidence. All of them work in high-level positions. They do what they’ve decided they want to do and make sure things go according to their wishes…But they’re very concerned with how things look from the outside: everything has to be planned before the baby is born, and that creates a kind of stress that isn’t always so good. Those who are very performance-oriented don’t feel good. They have problems setting limits for themselves and saying no to their bosses and others in their social circles. You have to help them reduce their workload and reassure [them] that it’s OK to do so.* (Participant, FGD no. 5).

#### Responding to perceived barriers to physical activity in pregnant women

It was perceived that most of the pregnant women did not prioritize physical activity. Several barriers to physical activity in terms of physical problems – tiredness due to the pregnancy, back pain, or pelvic pain were discussed. Other perceived barriers were lack of time, caring for children, or having a heavy workload:

*The multiparous women say they don’t have the time [to be physically active]. You know – dropping off and picking up children from day care, fatigue, a lot of things. So sometimes I find multiparous women are more difficult [to counsel] about their physical activity when I meet them.* (Participant, FGD no. 3).

The participants expressed that some pregnant women also exhibited psychological resistance towards physical activity because they may have had a history of failure of achieving their goals regarding physical activity. Other perceived obstacles to participating in physical activity included a sedentary lifestyle and lack of knowledge on how to perform physical activity. Some women simply preferred a sedentary lifestyle; these women viewed their pregnancy as a time when they did not have to exert themselves in voluntary physical activities.

*They’ve tried so many times already and are afraid of failing yet again. So perhaps they get stuck in this mind-set somehow. I’m sure that many would like help, but often other things are needed for them to have the strength to make a change in their lifestyles. Sometimes they seem to prefer living an unfulfilling existence to the risk of failing in something enjoyable.* (Participant, FGD no. 3).

*…to lie on the sofa and eat pralines just because it’s allowed. It’s almost like they’re making hay while the sun shines, because it’s somehow permissible now [when they are pregnant].* (Participant, FGD no. 2).

Low socio-economic status was perceived as associated with physical inactivity; participants working in low socio-economical areas stressed that many pregnant women had neither economical means nor previous experience with physical activity. Hence, some pregnant women would not even attend free exercise sessions that promoted and required physical activity designed specifically for pregnant women.

#### Responding to the cultural tug-of-war

For both the Swedish-born and the immigrant women, culture influenced how pregnant women were counselled. This tug-of-war of beliefs – resting versus physical activity – was characterized by contradictions between the messages conveyed by the participants, and the opinions and traditions expressed by the pregnant women and their relatives. It was challenging to explore pregnant women’s own wishes for physical activity during pregnancy in relation to her cultural traditions, as they often did not coincide. The participants discussed the importance of adjusting their recommendations of physical activity to conform to the wishes, often influenced by cultural expectations, of the pregnant women, although these recommendations did not comply with general recommendations in the guidelines.

*And many stop being active when they become pregnant because of quite severe pressure from relatives [who say] that it [physical activity] is dangerous and so on. So it becomes difficult for them to manage this issue during pregnancy, I feel. They stay home for the first three months and do nothing but lie on the sofa, sedentary.* (Participant, FGD no. 7).

Conditions, such as uncomfortable weather and lack of appropriate clothing, were other reasons for pregnant women to avoid physical activity. Some women were not used to outdoor activities in cold or rainy weather conditions. Others were afraid of getting lost when walking in unfamiliar areas. As such, these women experienced some social isolation due to these circumstances. Some other cultural situations made it difficult for women to be active. For example, some cultural expectations require women to be escorted by a male relative when out of the house, so participants had to consider cultural expectations when providing counselling:

*There are many who are afraid, who won’t go outside on their own, and there may be many reasons why they’re reluctant to do so. Many don’t know their way around, so I make a point of telling their husbands: “You need to walk with her on the same route several times so she gets to know the way. Then she can walk on her own.” Others tell me that they’ve never been out on their own, that they’re not allowed to do so. They have to go with a cousin, mother, brother, or someone else.* (Participant, FGD no. 4).

Some recommended physical activities, such as swimming in public pools, were not an alternative for some groups of immigrant women. When there was no other alternative for physical activity, the midwives recommended walking:

*If you suggest that they should go swimming, it could prove to be a snag because they aren’t allowed to swim at the same time as men. That’s one thing. The second is that they don’t know how to swim. There are swimming schools for women, but then they don’t have the money for that…There are so many barriers the whole time. So walking has become our mantra because it’s so beneficial and it is for free.* (Participant, FGD no. 4).

Personal suffering (such as depression, economical problems, health issues, and isolation) could be a barrier to physical activity during pregnancy. Some pregnant women had other problems to face, such as being a refugee with many other worries in their life. In these cases, counselling did not focus on physical activity:

*People have different priorities in life. If your life involves having migrated from somewhere else and you’re trying to adapt, then physical activity is not your first priority…* (Participant, FGD no. 4).

### Counselling as walking the thin ice

This category includes the sub-categories *Fearing no success, Guarding the relationship*, and *Acting upon individual needs* and addresses how participants experienced the situation of counselling as such. The three subcategories involve participants’ fear of failing to fulfill the counselling goals identified in recommendations and guidelines. Furthermore, the participants attempted to protect the relationship and to individualize counselling to each woman’s specific needs.

#### Fearing no success

The encounters were often considered as golden opportunities to promote lifestyle changes and to increase physical activity for most pregnant women. Fear of failure to provide appropriate counselling in relation to recommendations and guidelines while not being offensive often resulted in tiptoeing. Most expectant parents were perceived to have high expectations with respect to midwives’ professional performance, and the participants also expressed high expectations with respect to their performance as counsellors. Additionally, colleagues and physicians expected that the pregnant women would follow the midwives’ recommendations. If a pregnant woman voluntarily left one ANC clinic for another or one midwife for another, it was perceived as indication of professional failure. There were some situations when the midwives did not succeed in promoting any level of physical activity, leaving these midwives feelings frustrated, indifferent, and resigned:

*Sometimes you succeed. Sometimes you don’t and they remain inactive throughout their pregnancy no matter what you do…And some equate housework with physical activity and you try to explain that cleaning and cooking is not the same as taking a 30-minute walk…But, well…* (Participant, FGD no. 8).

#### Guarding the relationship

The participants were eager to safeguard the relationship and they wanted to ensure that the encounters were held in a positive atmosphere. To avoid overly pressuring the pregnant women, they slowly gave out advice, step-by-step. This strategy was especially important when helping sedentary women. In such cases, the midwives gradually introduced strategies to help encourage physical activity. To provide individualized counselling, the participants had to evaluate the woman’s life situation. This knowledge could suggest strategic decisions such as waiting for an appropriate time to discuss a problem:

*You want the encounters to be nice and enjoyable when they visit us. And if they’re overweight and perceive that we’re only focusing on these issues [diet and physical activity], then it will not be nice to see us, so you have to choose when to address the issues.* (Participant, FGD no 6).

#### Acting upon individual needs

Evaluating each woman’s situation was perceived as necessary for effective counselling on physical activity. This individualization was viewed as an on-going process during the course of the pregnancy. This on-going adjustment of strategies was used to meet the individual needs of each woman and was expressed in terms such as “intuitive assessment”, “tiptoeing”, and “being a chameleon”.

*Of course, it [counselling] is an individual thing because the women who come to us are so very different from one another. Some talk about certain things while others have such difficulties socially that there is no use in trying. They’re not in situations where diet and physical activity are appropriate topics for discussion. So, for me, there is a constant need to adapt, which makes this line of work both fascinating and difficult.* (Participant, FGD no. 3).

Being overweight or obese was perceived as a sensitive issue. Some of the pregnant women had been overweight since early childhood and had been exposed to a variety of advice from other health care providers, advice that had not always been received positively. Although being overweight was considered a sensitive topic to address, some of the participants felt that information concerning maternal weight should be delivered frankly; these participants thought too much tiptoeing or completely ignoring the weight issue was unprofessional:

*Of course she knows she’s overweight; it would just be silly not talking about it or pretending she’s not.* (Participant, FGD no. 1).

Some participants felt that their own lifestyle and body shape might negatively influence the counselling. For example, some participants thought that their fitness (i.e., their physical appearance with respect to their perceived body shape) influenced how the pregnant women received their advice on physical activity. However, the majority of participants did not agree with this perspective as they believed counselling should be performed in a professional manner irrespective of the midwife’s own lifestyle and perceived fitness.

### Counselling as an opportunity

This category includes the two sub-categories *Navigating within cultural traditions* and *Identifying women’s’ individual facilitators*, subcategories that highlight the midwives’ experiences and strategies navigating cultural concerns and identifying and using facilitators that would help individual women participate in appropriate physical activity.

#### Navigating within cultural traditions

Despite challenges due to cultural traditions, the participants tried to comprehend the situation and adjust their counselling based on their cultural understanding, looking for culturally acceptable alternatives for physical activity. A major part of counselling consisted of informing these women on the significance of physical activity during pregnancy, assessing possible forms of physical activity, and assuring them that physical activity for most pregnant women would be safe and beneficial.

*Working with immigrant women is so much harder; to make them believe what I’m saying. I find it easier to convince educated women… But this issue [not listening to advice] seems to be influenced by culture and, in my eyes; we have a very long way to go before [our counselling] meets with a response.* (Participant, FGD no. 4).

Cultural or psychosocial concerns also required participants to adjust the recommended physical activity goals. The desired levels of physical activity during pregnancy were lowered in order to reach some level of physical activity during pregnancy that was acceptable for the pregnant woman.

*That they make an effort at all, even if it’s not…That is to say, they don’t have to aim for the stars. A short walk is good enough.* (Participant, FGD no. 4)*.*

#### Identifying women’s individual facilitators

The participants tried to identify each woman’s individual motivational factors for physical activity. General factors motivating physical activity were strengthening of maternal health during pregnancy, avoiding extensive gestational weight gain, and maintaining a healthy foetus. It was easier to motivate pregnant women for physical activity if they had been physically active before their pregnancy. In those cases, counselling often addressed suitable forms and activity levels of physical activity instead of promoting increases in activity.

*There are women who discovered this [wellbeing as a result of physical activity] before their pregnancy. They are more motivated … And it’s easier to motivate someone who was already exercising beforehand.* (Participant, FGD no. 8).

*This [identifying individual motivators] is important and, in such cases, you need to be incredibly perceptive too. What will their situation be when they come in today? What is their starting point? What motivates them? And then, [for me to] provide as much help as possible.* (Participant, FGD no. 6).

## Discussion

This study investigated midwives’ experiences with counselling pregnant women on physical activity during pregnancy. The findings show that counselling pregnant women about appropriate levels of physical activity was perceived as multifaceted. The participants identified barriers and promoted facilitators. As the participants considered these topics sensitive for some women, especially overweight and obese women, they were concerned that their counselling could offend some women to such an extent that their advice would be ignored. These demands added to the stress they already felt performing their other professional duties. The theme *“An on-going individual adjustment”* describes how the participants repeatedly assessed pregnant women’s barriers and facilitators to physical activity, adjusting their counselling accordingly. Even small improvements in physical activity were acceptable when the participants realized the extent of barriers – cultural or individual – some women had to face. The participants reported a number of perceived barriers – tiredness, lack of time, and bodily pain – pregnant women had to overcome to perform physical activity. Similar barriers for performing physical activity have been described previously [10, 11, 16].

Achieving desired behavioral change in pregnant women often requires giving the women themselves a central role in the antenatal care; such an approach requires a good relationship between the midwife and the pregnant women [5, 18]. A mutually trusting relationship is important when counselling women with gestational diabetes mellitus [5], but some studies report that intensive counselling on physical activity using interventions may be associated with a decrease in physical activity during pregnancy [19, 20]. Similarly, midwives have reported resistance when counselling pregnant women about cessation of smoking, also a case where maintaining a good relationship with the women is important for a successful outcome [21]. Our findings highlight the significance of individual adjustments of counselling based on the on-going assessment and evaluation of a pregnant woman’s individual needs and situation. Similar results are demonstrated when investigating health care providers’ counselling on diet and gestational weight gain, where assessment of individual needs is important for improving changes in lifestyle [22]. Furthermore, the participants noted that financial cutbacks have negatively affected the quality of their counselling.

The participants found it challenging to provide effective counselling to women whose participation in physical activity was limited by cultural expectations. Similar results have been reported in studies exploring maternity care assistants and midwives providing postpartum care [23, 24]. These studies, performed in a Dutch setting, describe how the work with immigrant women needs to be flexible and creative, especially addressing issues related to lack of financial resources, low socioeconomic status, and family pressure. Individualized solutions for these women have been shown to improve the quality of the health care [23, 24]. Studies addressing weight gain during pregnancy have shown that midwives’ counselling may not have the desired impact on pregnant women from other ethnic backgrounds since these women may be more influenced by the traditions in their own culture and by the views of their relatives [25, 26]. Our study’s findings agree with these assessments.

Few studies have considered whether midwives’ own body shape and lifestyle influence how they counsel pregnant women. When providing health promotion advice, overweight and obese nurses have to deal with the fact their patients may perceive their advice as insincere [27]. In our study, some participants did not believe that their own body shape would affect their counselling; however, others reported that this could influence their counselling both in a negative and in a positive way. These findings are consistent with a study exploring overweight nurses’ experiences with overweight non-pregnant patients; some overweight nurses believed their body shape helped them connect and empathize with their patients, although some did not [28]. In our study, some participants felt uncomfortable discussing their own body shape during the FGD. Similarly, some participants were afraid to directly address individual patient’s weight issues, tiptoeing around the issue. These findings agree with findings in previous studies where excessive weight gain and obesity during pregnancy were perceived by health care providers as sensitive subjects to discuss [14, 24, 25].

Our study’s findings have some clinical implications. Additional formal education and in-service training for midwives should be provided so midwives have the tools and strategies to use when providing counselling of complex situations and living conditions. A meta-analysis evaluating the efficacy of motivational interviewing (MI) in 48 studies in medical care settings reveals that using MI may be beneficial in a number of areas, for example, improving sedentary behavior [29]. Of course, more education and extensive training means that organizational barriers such as time restraints and economical cutbacks need to be addressed. However, the Swedish National Board of Health and Welfare suggests that counselling about lifestyle changes, such as increasing physical activity, should be performed in short sessions of 10-15 minutes and for more challenging cases, 30 minutes [30]. It is unknown whether this fairly new recommendation was known and implemented by the midwives participating in our study. As 10-15 minutes is a rather short time, it is also difficult to know if “time restraint” is used as an excuse for not prioritizing counselling about physical activity.

Future studies should investigate counselling about physical activity in relation to cultural issues. In addition, future studies should address whether and how health care professionals’ own body shape and lifestyle influences their work as counsellors. Individual in-depth interviews may address this research question, as such sensitive topics may be difficult to discuss in a group setting.

### Methodological considerations

A strength of this study is the purposive sampling where the participants represented varying characteristics in relation to age, length of working experience, and geographical regions with different socio-economical status. To ensure transferability of the findings, the data collection had no pre-set number of FGD. As a result, data collection continued until no major new information regarding the topics under study was expressed (i.e., additional interviews would not have provided major new information). Additionally, a rich description of the procedure and analysis was provided to enable the reader to follow all steps of the research process. To increase credibility of study findings, member checks were performed, which confirmed the findings. Writing memos and keeping records of the process of data collection and analysis addressed dependability of the findings. Moreover, the same persons from the research team performed all FGD, which also contributed to increase dependability. Finally, the confirmability of the findings was achieved through discussing the findings within the research team until consensus was obtained. As members of the team had different professional backgrounds, objectivity was enhanced as individual preunderstandings could be challenged.

Despite these efforts to address trustworthiness, there are some limitations in the study. The participants were initially approached and informed about the study by the consultant midwives in their MHCA. Although these consultant midwives were given detailed information on the purpose of the study and the inclusion criteria, we cannot exclude that the consultant midwives chose convenient participants irrespective of this information.

## Conclusions

Counselling on physical activity during pregnancy may be a challenging task for midwives, characterized by on-going adjustments based on a pregnant woman’s individual needs. Midwives strive to find individual solutions to encourage physical activity. However, to improve their counselling, midwives may benefit from further training, so organizational and financial barriers need to be addressed. Such efforts might result in improved opportunities to further support pregnant women’s motivation for performance of physical activity.

## Authors’ information

The authors represent various professional disciplines and research traditions including midwifery, obstetrics and gynaecology, physiotherapy, nursing, and public health.
